# Quantifying indices of short- and long-range white matter connectivity at each cortical vertex

**DOI:** 10.1371/journal.pone.0187493

**Published:** 2017-11-15

**Authors:** Maria Carmela Padula, Marie Schaer, Elisa Scariati, A. Kadir Mutlu, Daniela Zöller, Maude Schneider, Stephan Eliez

**Affiliations:** 1 Developmental Imaging and Psychopathology Laboratory, University of Geneva School of medicine, Geneva, Switzerland; 2 Neuro-Electronics Research Flanders, Leuven, The Netherlands; 3 Medical Image Processing Laboratory, Institute of Bioengineering, Ecole Polytechnique Fédérale Lausanne (EPFL), Lausanne, Switzerland; 4 Department of Genetic Medicine and Development, University of Geneva School of medicine, Geneva, Switzerland; University of Pennsylvania, UNITED STATES

## Abstract

Several neurodevelopmental diseases are characterized by impairments in cortical morphology along with altered white matter connectivity. However, the relationship between these two measures is not yet clear. In this study, we propose a novel methodology to compute and display metrics of white matter connectivity at each cortical point. After co-registering the extremities of the tractography streamlines with the cortical surface, we computed two measures of connectivity at each cortical vertex: the mean tracts’ length, and the proportion of short- and long-range connections. The proposed measures were tested in a clinical sample of 62 patients with 22q11.2 deletion syndrome (22q11DS) and 57 typically developing individuals. Using these novel measures, we achieved a fine-grained visualization of the white matter connectivity patterns at each vertex of the cortical surface. We observed an intriguing pattern of both increased and decreased short- and long-range connectivity in 22q11DS, that provides novel information about the nature and topology of white matter alterations in the syndrome. We argue that the method presented in this study opens avenues for additional analyses of the relationship between cortical properties and patterns of underlying structural connectivity, which will help clarifying the intrinsic mechanisms that lead to altered brain structure in neurodevelopmental disorders.

## Introduction

Diffusion tensor imaging (DTI) provides a powerful method to examine patterns of white matter connectivity [1, 2]. Initial analyses used DTI to compute voxel-based measures of white matter integrity, such as fractional anisotropy (FA) or axial (AD) and radial diffusivity (RD), which reflect axonal organization and myelination [3, 4]. More recently, tractography has been proposed to reconstruct the pathways of fiber tracts [5] and compute the structural “connectome” of the brain [6, 7]. Quantitative comparisons of the white matter properties can be conducted either by measuring the number of streamlines connecting different cortical regions [8–12] or by quantifying the structure of the connectomes using measures of graph theory [13].

The application of current DTI methods in healthy and clinical populations provided insights about the normal and pathological patterns of white matter connectivity. However, these techniques present some problems, mostly related to registration issues. Recent studies indicated that volume-based registration techniques are less reliable than surface-based methods [14, 15]. The inaccuracy of volume-based registration algorithms is even more evident in clinical populations, where alterations in individual brain morphology impair the optimal matching with templates obtained from healthy populations [16, 17]. In turn, inaccurate registration can lead to imprecisions when comparing voxel-based metrics of fibers bundles’ integrity between subjects [18]. Such a registration bias is usually less evident with tractography reconstructions, where regions of interest (ROIs) can be defined on the cortical surface. However, the a priori defined ROIs used to generate connectivity matrices are typically based on atlases composed of large brain regions that may encompass distinct functional areas [19, 20]. To overcome this issue, a number of investigations proposed methods to increase the resolution of the connectome reconstruction by increasing the number of ROIs (see for instance [6, 21, 22]). These studies showed that higher resolution reconstructions were reliable, but characterised by greater inter-subjects variability, which can affect the accuracy of group comparisons. In sum, it remains challenging to provide metrics of altered white matter connectivity that are not biased by registration issues or not restrained within anatomically constrained regions.

In this study we propose an approach to obtain metrics of white matter connectivity in the cortical surface space. We believe that the projection of the fiber tracts to the cortex would provide several advantages: 1) The use of well-validated inter-subject surface-based registration algorithms [23, 24], which improve statistical power and ability to detect group differences; 2) the computation of more fine-grained metrics of connectivity, unconstrained by anatomical boundaries; and 3) a more easily interpretable visualization of the patterns of connectivity over the cortex.

Several recent studies proposed innovative approaches to combine brain morphology and white matter connectivity [25–30]. In particular, in [26], the authors used a similar approach to represent the connectivity information as a continuous measure over the cortical space. The authors defined connectivity metrics, indicating the percentage of connections from one lobe to another, in the native space of each subject, using an original approach relying on a surface-based quantification of connectivity. In [30] the authors defined indices reflecting the proportion of short- and long-range connections and their development with age. Even if innovative, both approaches were still based on the a priori definition of ROIs, thus preventing a high-resolution visualization of white matter over the cortex.

In the present work, we propose an alternative method to map the white matter tracts in the cortical space and interrogate connectivity patterns from any cortical vertex using a convenient visualization. Our method is not based on the reconstruction of a connectivity matrix, but streamlines are generated and their extremities (starting and ending points) are mapped on the cortical surface, as reconstructed with *FreeSurfer*, and attributed to a cortical vertex. Therefore, the metrics we propose can be used for vertex-wise comparisons in the cortical space, using reliable surface-based registration methods [24], further allowing for an intuitive interpretation of the statistical group differences over the cortical surface. Another advantage of our methodology is that it complements extant measures of cortical morphology, such as thickness and gyrification, and functional brain activation, thus representing an optimal tool for multimodal investigations of brain connectivity.

The metrics that we propose in this study summarize information about average connection length, as well as measures of short- and long-range connection patterns. The distinction of short- and long-range connectivity is important as it reflects different processes in the brain, namely segregation and integration. Segregation is defined as the specialization of a brain area to accomplish a specific function, therefore it is a process thought to be subserved by local connections [31, 32]. Integration reflects instead the communication between distant brain areas throughout long-range connections [31] and is essential for ensuring high order cognitive functions such as visual recognition, language, cognitive control and social cognition [32]. Furthermore, these measures change through development with short-range connectivity decreasing and long-range connectivity increasing, thus reflecting decreased segregation and increased integration [33–36].

To examine the potential of our method to quantify meaningful differences in connectivity patterns, we used a clinical dataset of 62 patients affected by 22q11.2 deletion syndrome (22q11DS) and 57 typically developing individuals. 22q11DS is a neurogenetic disorder that puts affected individuals at high-risk of developing schizophrenia [37, 38]. The common phenotype of patients with 22q11DS includes a characteristic facial appearance, cardiac defects [39], cognitive impairments and psychiatric manifestations [38, 40–43]. Extant neuroimaging studies have delineated the patterns of alterations in brain morphology [44–49] and structural connectivity [50] in the syndrome. In particular, these studies reported reduced efficiency [51] and reduced long-range connections [10, 50] in patients with 22q11DS, thus suggesting reduced integration. Furthermore, the brain phenotype associated to 22q11DS includes polymicrogyria [52], and reduced cortical gyrification has largely been reported in patients with 22q11DS [53–57]. A previous study using indices of short-range connectivity showed reduced short connections in individuals with polymicrogyria [58]. Altered short-range connectivity has also been reported in patients with other developmental disorders such as autism [36, 59, 60] and schizophrenia [36], and it has been suggested to be associated to impaired synaptic pruning.

Therefore, we expect that our measures would better capture alterations in both short- and long-range connections in patients with 22q11DS, providing a more precise localization of the alterations over the cortical surface.

## Materials and methods

Written informed consent was received from all the subjects and their parents using protocols approved by the cantonal ethic commission of research.

### Method overview

**Fig 1:**
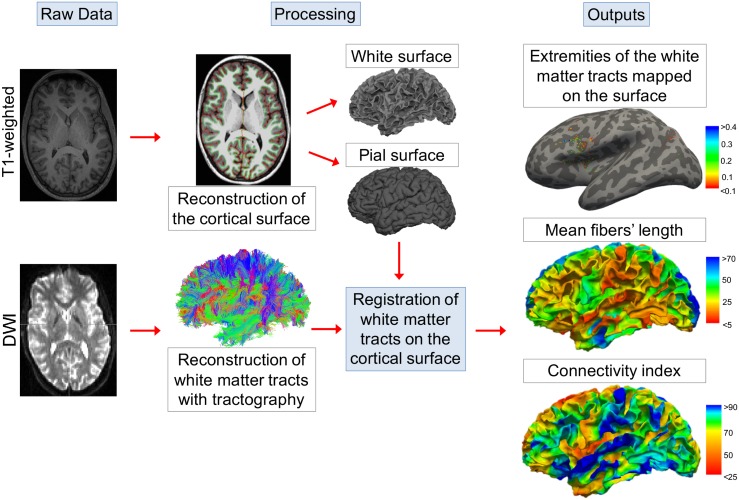
Overview of the method used to compute connectivity measures on the cortical surface. T1-weighted and diffusion weighted images (DWI) were used to reconstruct the cortical surface and the white matter bundles, respectively. The tracts were then registered to the cortical surface space. The mean fibers’ length was computed as the mean length of the fibers starting from each vertex. The connectivity index was defined as the ratio between the number of short or long fibers over the total number of fibers starting from the vertex.

**Fig 2:**
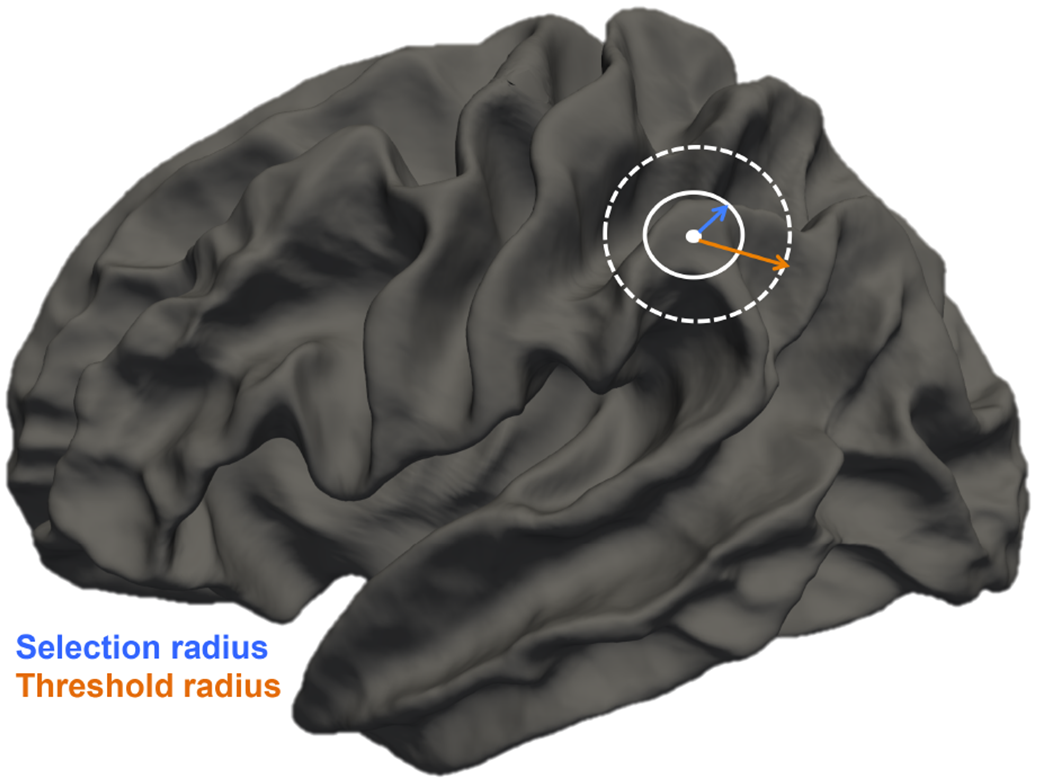
Computation of the connectivity index (CI). The point indicates the starting vertex, while the blue and the orange arrows represent the selection and the threshold radius, respectively. The CI was defined as the ratio between the number of fibers ending in the threshold sphere over the total number of fibers starting in the selection sphere. The indices of short- and long-range connectivity are obtained by using different threshold radii ranging from 5 to 100 mm.

The purpose of the present study was to compute fine-grained measures of connectivity that could be displayed on the cortical surface. Fig 1 summarizes the information flow of our method. Briefly, T1-weighted images were used to reconstruct three-dimensional cortical surfaces. In parallel, tractographic reconstruction of the white matter bundles was performed using the diffusion weighted (DWI) scans. The fiber tracts were then registered to the space of the cortical surface. For each cortical vertex, we selected the fibers that had at least one of their extremities within a 5 mm radius. The selected fibers were then used to compute 2 measures of connectivity for each cortical vertex: 1) the mean tracts’ length, and 2) the connectivity index (CI), a ratio that provides information about the proportion of short- or long-range connections over the cortical surface (see also Fig 2).

### 0.1 Reconstruction of the cortical surface

The anatomical T1-weighted images were processed using the *FreeSurfer* software (http://surfer.nmr.mgh.harvard.edu) to produce accurate 3D mesh models of the cortex in the native space of each subject. The surface reconstruction process consists of previously validated steps [23, 61], including resampling into cubic voxels, intensity normalization, skull stripping, tissue segmentation and tessellation of the cortical surfaces. At the end of this procedure, cortical surfaces were obtained at the boundary between the white and gray matter (white surface), and at the boundary between the gray matter and cephalo-spinal fluid (pial surface).

### 0.2 Tractography

Fiber tracts were estimated from the DWI acquisition using different tools embedded in the Human Connectome Mapper toolkit (http://connectomics.org, [62]). First, DWI images were corrected for the effect of head motion and distortion of eddy currents using FSL (http://fsl.fmrib.ox.ac.uk/fsl/fslwiki/EDDY). T1-weighted images were then registered in the diffusion space using a boundary-based registration implemented in *FreeSurfer* and FSL (https://surfer.nmr.mgh.harvard.edu/fswiki/bbregister). Then, tractography was computed using a deterministic streamline algorithm provided with the camino software (http://camino.cs.ucl.ac.uk). Default parameters were used for the streamlines reconstruction (curvature threshold = 60, number of iterations 50) and a white matter mask obtained from the freesurfer processing was used as seed. Two tensors were modelled at each voxel of the white matter mask obtained from the *FreeSurfer* pipeline, and white matter streamlines were propagated voxel by voxel until both ends reached the grey matter mask. As the number of reconstructed streamline is proportional to their length we corrected this bias using a method described in previous studies [63, 64]. In particular, we attributed to each streamline a weight equal to the inverse of its length.

### 0.3 Connectivity measures in the cortical surface

After reconstruction, streamlines shorter than 3mm were removed and the remaining tracts were registered to the cortical space using the transformation matrix computed above. For each cortical vertex, we selected all streamlines that had one extremity within a 5 mm radius from this vertex (“selection radius”). Hereafter, we refer to those fibers as “starting from the vertex”. This definition is used only for clarity purposes, as the fiber tracts obtained with DTI do not have any directionality. At this stage, it was possible to visualize where the streamlines starting from each vertex ended on the cortical surface. The “ending point” of the fiber was defined as the vertex closest to its other extremity. At each vertex, the mean tracts’ length was computed as the mean length of all fibers starting from this point. The connectivity index was computed as the ratio of the amount of short- or long-range fibers starting from the vertex divided by the entire number of streamlines starting at that point (Fig 2). Specifically, the *CI*_*short*_ (short-range connectivity index) was defined as:

$$\begin{array}{c} CI_{short}=\frac{n.\;short\;fibers}{tot.\;n.\;fibers}*100 \end{array}$$ (1)

where “n. short fibers” represents the number of short-range streamlines and “tot. n. fibers” is the total number of streamlines starting from the vertex. Similarly, the *CI*_*long*_ (long-range connectivity index) was defined as:

$$\begin{array}{c} CI_{long}=\frac{n.\;long\;fibers}{tot.\;n.\;fibers}*100 \end{array}$$ (2)

where “n. long fibers” represents the number of long-range streamlines starting from the vertex.

In the absence of any clear consensus about the definition of short- and long-range connections [60, 65–67], we decided to use the average mean tracts’ length measured in our group of subjects (30 mm) as the cut-off. Thus, short fibers were defined as streamlines with a length ≤ 30 mm, long fibers as fibers with length ≥ 30 mm. Given that this threshold can be considered arbitrary, we further tested the behaviour of our algorithm at different thresholds, from 5 to 30 mm for the *CI*_*short*_ and form 30 to 60 mm for the *CI*_*long*_, with steps of 5 mm. Statistical comparisons were computed at each threshold but for simplification purposes the cortical significance maps are shown here for one threshold only: 20 mm for the short-range and 60 mm for the long-range connectivity indices.

Reliability and inter-subject variability tests have been performed for both our indices using the Intra Class Correlation and the Coefficient of Variation. The methods and results of this analysis have been reported in Supporting Information (S1 and S2 Figs).

### 0.4 Application in a clinical sample

The method was tested in a group of 62 patients affected by 22q11.2 deletion syndrome (22q11DS) and 57 control participants. The patients with 22q11DS were aged from 6 to 28 years old (mean age = 15.7±5.2; 30 males) and their mean IQ was 67.5±10.6. The presence of a 22q11.2 microdeletion was confirmed using quantitative fluorescent polymerase chain reaction. The control participants were aged from 6 to 28 years old (mean age = 17.3±5.7; 26 males) with a mean IQ of 106.4±12.3. Participants’ handedness was assessed using the Edinburgh laterality quotient [68]. The proportion of right-handed did not significantly differ between the control and the patient groups (74% of right-handed in the control group and 77% in the patients, *χ*^2^ = 0.225, p = 0.635).

In this sample of participants, T1-weighted and diffusion tensor images were acquired using a Siemens Trio 3 Tesla scanner at the Geneva Center of Biomedical Imaging (CIBM). The anatomical sequence had the following parameters: TR = 2500 ms, TE = 3 ms, flip angle = 8°, acquisition matrix = 224 x 256, field of view = 220 mm, slice thickness = 1.1 mm, 192 slices. The diffusion weighted imaging scans were acquired using the following sequence: number of directions = 30, b = 1000 s/mm^2^, TR = [8300–8800] ms, TE = 82 ms, flip angle = 90°, acquisition matrix = 128 x 128, field of view = 25.6 cm, slice thickness = 2 mm.

### 0.5 Statistical analysis

In order to compare the mean tracts’ length and the CIs between groups, the cortical surface of each subject was registered to an average spherical surface [24]. Data were then resampled to the common average spherical coordinate system and smoothed using a nearest-neighbor averaging procedure with a full-width at half-maximum (FWHM) of 10 mm. Statistical differences between groups were evaluated using a general linear model (GLM) with the Query Design Estimate Contrast (QDEC) interface of *FreeSurfer*, including age and gender as covariates. Given the previously reported reduced white matter volume in patients with 22q11DS [69, 70], we repeated the analysis taking white matter volume into account in the model. Montecarlo multiple comparisons correction was performed for the mean tracts’ length and at each threshold of the short and long connectivity indices. To provide robust protection against type 1 errors, we used a stringent cluster wise p-value threshold at *p*<0.01 [71].

## Results

### 0.6 Group differences in the mean tracts’ length

**Fig 3:**
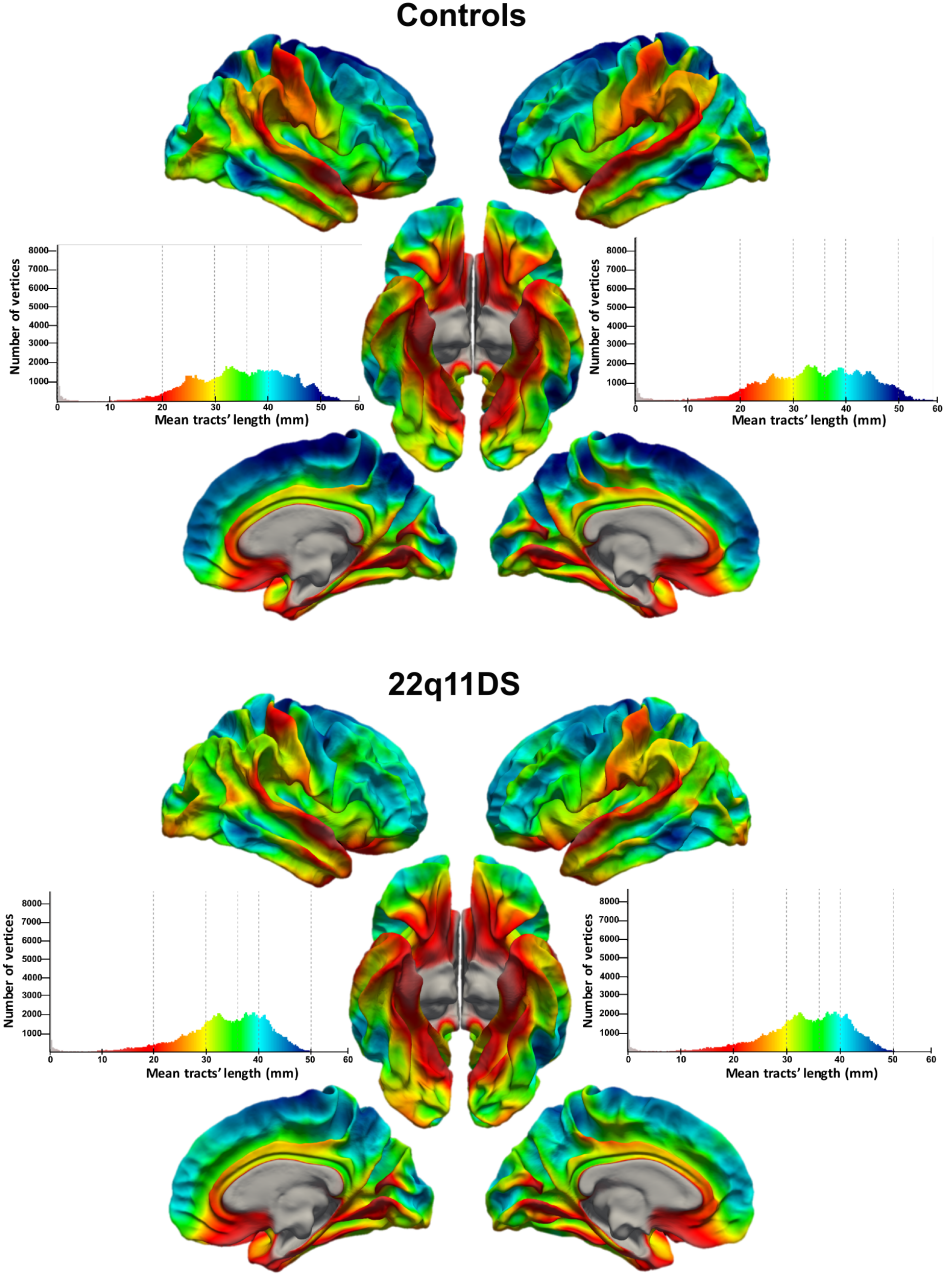
Cortical maps of the mean tracts’ length and corresponding histograms. The maps represent the average measures of mean tracts’ length for each group. The dorsomedial cortical regions had longer streamlines, followed by ventromedial prefrontal regions, post-central and prefrontal cortices.

Fig 3 depicts the average mean tracts’ length for each cortical point in the group of controls and patients with 22q11DS. In both groups, longer streamlines (blue color) were located in the dorsal medial frontal and parietal cortices and in the lateral superior and middle frontal cortices. Local minima in the mean tracts’ length (red color) were instead observed in postcentral and superior temporal cortices as well as in orbitofrontal and inferior temporal regions.

**Fig 4:**
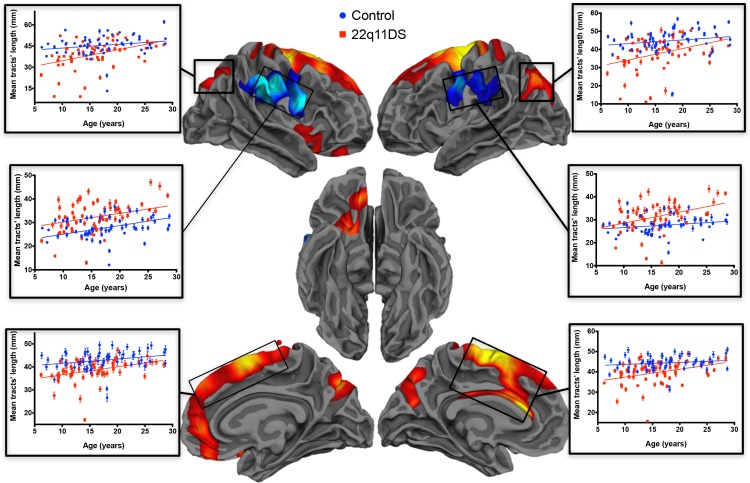
Clusters of significant between-groups difference in mean tracts’ length. Most of the clusters of decreased mean fibers’ length in patients compared to controls (yellow/red) were symmetrical. Only one cluster of increased mean tracts’ length (blue scale) was found, in the bilateral pre/post central cortices spanning trough the supramarginal gyrus.The plots represent the values of average length in each cluster.

As shown in Fig 4 and Table 1, the clusters where the mean tracts’ length was significantly reduced in patients with 22q11DS were mostly symmetrical in both hemispheres and were located in the inferior parietal cortex extending medially to the precuneus and in dorsal medial frontal regions including the left cingulate cortex. One cluster of significantly increased mean tracts’ length was found in 22q11DS, comprising the lateral pre and postcentral cortices and spanning trough the supramarginal gyrus. When including white matter volume as a covariate in the model, the differences in the left hemisphere remained significant in all previously found regions except for the anterior cingulate cortex, while in the right hemisphere only the pre/postcentral cluster remained significant (see also the details in Table 1).

**Table 1:** Clusters with between-groups statistical difference in mean tracts’ length.

Region	Difference	Cluster size	Peak coordinates (x y z)	Cluster wise *p*	Effect size
**Left**					
Anterior cingulate cortex	Contr>22q11	6754 *mm*^2^	-1.5 24.3 15.4	0.0001	0.6773
Superior parietal cortex*	Contr>22q11	3570 *mm*^2^	-22.6 -65.7 29.4	0.0001	0.6404
Precentral cortex*	22q11>Contr	3344 *mm*^2^	-50.1 -7.6 37.4	0.0001	-0.6034
**Right**					
Supramarginal gyrus*	22q11>Contr	5463 *mm*^2^	52.9 -28.9 40.2	0.0001	-0.7207
Precentral/superior frontal cortex	Contr>22q11	8436 *mm*^2^	16.8 -13.6 61.2	0.0001	0.6292
Precuneus	Contr>22q11	2034 *mm*^2^	14.1 -70.5 40.2	0.0003	0.6169

The * indicates that the difference remained significant after covarying for total white matter volume. Peak coordinates are given in Talairach coordinates, in mm.

**Fig 5:**
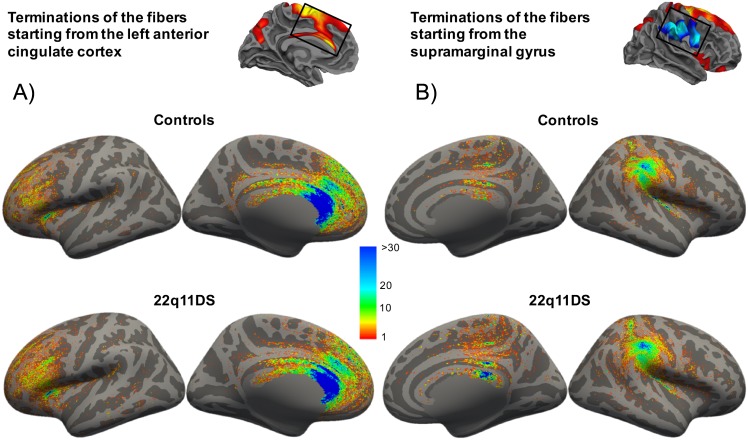
Terminations of the tracts starting from the vertices of maximally reduced and increased mean tracts’ length. In 22q11DS an increased proportion of connections from the anterior cingulate cortex ended locally in the inferior frontal cortex, while a decreased proportion of fibers ended distantly in the precuneus and the posterior part of the corpus callosum (A). On the opposite, an increased proportion of fibers starting form the supramarginal gyrus crossed the corpus callosum in patients with 22q11DS (B). To better visualize where the terminations of the streamlines were located on the cortex they were displayed on the inflated surface. The color bar indicates the proportions of terminations.

To better understand the altered patterns of white matter connectivity driving the observed differences in mean tracts’ length, we examined the topology of the fibers starting at the vertices of maximal between-group differences. Fig 5 illustrates the terminations of the fibers starting at the vertex of peak significance for the cluster located in the left dorso-medial prefrontal and anterior cingulate cortex (panel A), where we observed reduced mean tracts’ length, and the cluster in the right pre/postcentral cortices and supramarginal gyrus (panel B), where we observed increased mean tracts’ length in 22q11DS. In the control group (Fig 5A, top row), the fibers starting in the dorso-medial prefrontal/anterior cingulate cortex ended mostly locally in the same region as well as in the posterior cingulate cortex, genu, body and splenium of the corpus callosum, inferior, superior and medial frontal cortex, orbitofrontal cortex, precuneus, superior temporal sulcus. These results are in agreement with the previously reported connectivity patterns of the anterior cingulate cortex [72–74]. In the group of patients with 22q11DS (Fig 5A, bottom row), the localization of the terminations was almost overlapping except for an increased number of local connections with the inferior frontal cortex and a reduced number of long-range connections to the precuneus and the posterior part of the corpus callosum. This observation suggest that the reduced mean fibers’ length observed in the anterior cingulate cortex in patients with 22q11DS may be related to a reduced number of long-range connections starting from this region. We also plotted the terminations of the fibers starting at the vertex of peak significance for the cluster in the right pre/postcentral cortices and supramarginal gyrus (Fig 5B). In agreement with previous studies [75–77], the fibers starting from the vertex in the supramarginal gyrus ended mostly locally and in the central and postcentral sulci, inferior frontal cortex, insula, superior temporal cortex, splenium of the corpus callosum, posterior cingulate cortex, precuneus and medial paracentral cortex in both control individuals (Fig 5B, top row) and patients with 22q11DS (Fig 5B, bottom row). However, the amount of streamlines terminating in the splenium of the corpus callosum and the posterior cingulate cortex was higher in patients with 22q11DS than in controls. This evidence suggest that the increased mean path length observed in the superior temporal cortex in 22q11DS may in part rely on the increased number of long-range inter-hemispheric connections.

From the cortical maps in Fig 5, we observe that the majority of the terminations are located in sulcal rather than gyral regions. While this can seem contradictory with previous evidence showing that white matter tracts preferentially end in gyri [78], it should be noted that the maps presented in Fig 5 represent a subsample of fibers that start in a specific region, and not all the reconstructed fibers’ tract. In Supporting Information, we show that most of the reconstructed streamlines terminate in gyri (S3 Fig), consistently with previously published results [78].

### 0.7 Group differences in short- and long-range connectivity

**Fig 6:**
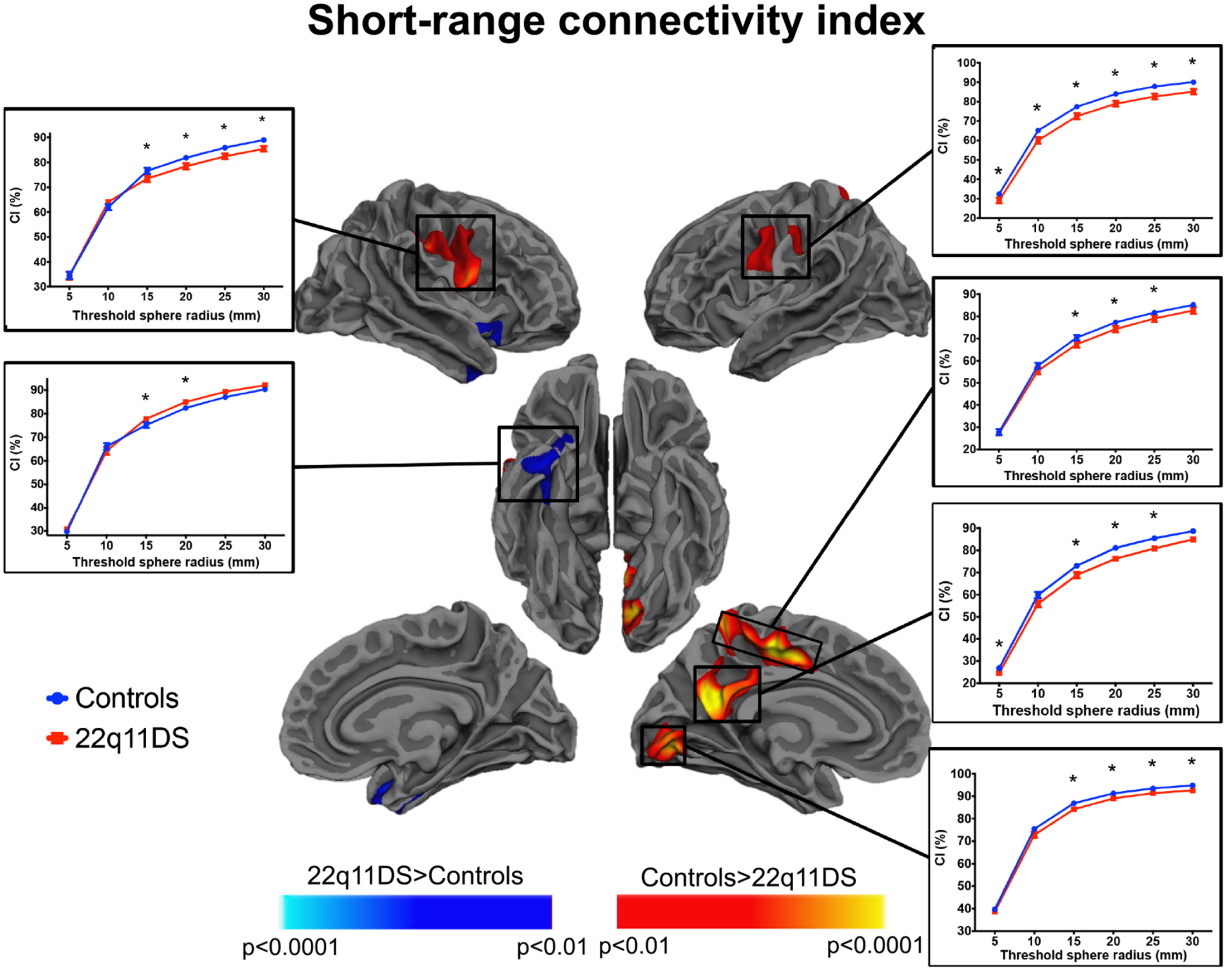
Between-group differences in short-range connectivity. The cortical maps represent statistical significance at p<0.01 (corrected) using a threshold of 20 mm to define the proportion of short-range connections. The plots, further depict values and significance of the *CI*_*short*_ at different thresholds ranging from 5 to 30 mm. We observed mainly clusters of reduced short connectivity in patients compared to controls (red/yellow). The cluster of increased *CI*_*short*_ (blue scale) in patients did not remain significant after correcting for the white matter volume. The * indicate that the *CI*_*short*_ significantly differed between controls and 22q11DS participants at that threshold.

**Fig 7:**
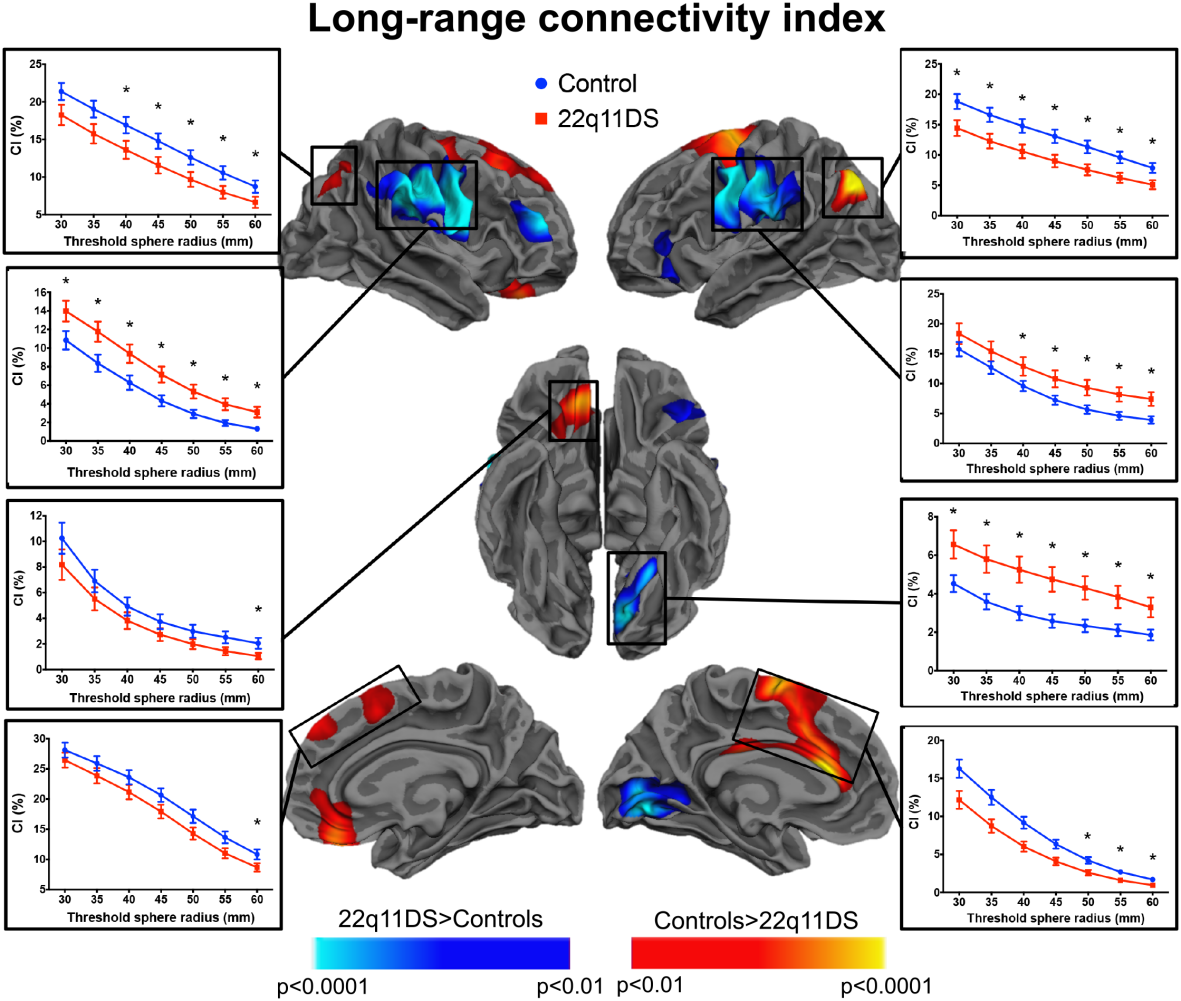
Between-group differences in long-range connectivity. The cortical maps represent statistical significance at p<0.01 (corrected) using a threshold of 60 mm to define the proportion of long-range connections. The plots, however, further depict values and significance of the *CI*_*long*_ at different thresholds ranging from 30 to 60 mm. The clusters of increased and decreased long-range connectivity mostly recapitulated the topology of the between group differences in mean tracts’ length. The * indicate that the *CI*_*long*_ significantly differed between controls and 22q11DS participants at that threshold.

Cortical maps showing significant between-group comparison in short-range connectivity are depicted in Fig 6, and maps of significant long-range connectivity differences are shown in Fig 7. As explained in the method section, the results are shown for the proportion of fibers shorter than 20 mm for the short-range CI, and for the proportion of fibers longer than 60 mm for the long-range CI. However, to further verify how the indices change as a function of the threshold, we display in the plots the values of *CI*_*short*_ and the *CI*_*long*_ for each threshold from 5 to 30 mm and from 30 to 60 mm, respectively.

As illustrated in Fig 6, we found clusters of reduced proportion of short fibers in patients compared to controls around the inferior region of the precentral gyrus bilaterally and in the left precuneus, calcarine and paracentral/posterior cingulate cortices. In addition, we also found one cluster of increased short-range connectivity in the left right anterior inferior temporal cortex, but this cluster of increased *CI*_*short*_ did not remain significant after covarying for total white matter volume.

As shown in Fig 7, reduced long-range connectivity in patients was observed bilaterally in the parietal cortex, and the dorso-medial frontal cortex spanning trough the anterior cingulate cortex in the left hemisphere and the right medial orbito-frontal/anterior cingulate cortex. We also evidenced clusters of increased long-range connectivity in patients compared to controls in the bilateral pre/postcentral cortex including the supramarginal gyrus, the left lingual cortex and the right middle frontal cortex. Only the clusters of increased CI remained significant after covarying for the total white matter volume. In summary, the patterns of changes in long-range connectivity mostly recapitulated the clusters where we observed significant between-group differences in the mean tracts’ length.

## Discussion

In this paper, we proposed an alternative method to compute, display and compare measures of white matter connectivity in the cortical surface’s space. We argue that our method, which leverages on the best surface-based registration techniques, provides a more reliable inter-subject registration compared to classical voxel-based registration techniques traditionally used in most DTI studies [3–7]. With measures at each vertex, we also achieved a more fine-grained resolution of connectivity indices over the cortex compared to existing methods. Studies trying to increase the resolution of the connectivity information over the cortex used an alternative approach, based on reducing the dimension of the nodes and building a high resolution connectome [6, 21, 22]. In this study, we decided to map the white matter information on the cortical space and obtain a measure of connectivity at each vertex. Our metrics do not carry the information about the number of fibres connecting pairs of brain regions, but they provide connectivity metrics for ∼ 150’000 vertices over the cortical surface. Using the example of 22q11DS, we discuss in the next sections how our method can help revealing important aspects of connectivity related to the patterns of short- and long-range connections, which are useful for a better characterization of neurodevelopmental diseases. Finally, we argue that, in a developmental framework, the projection of the connectivity information on the cortical surface will be critical to shed light on the relationship between cortical morphology and white matter connectivity.

Previous investigations using tractography to reveal white matter alterations in patients with 22q11DS mostly found reduced long-range connections [50] and reduced efficiency [51], both suggesting altered brain integration in these patients. Integration reflects impaired communication between distant brain areas [31], is essential for high order cognitive processes [32], and has been showed to develop with age, accompanied by a reduction in brain segregation [33–35]. We hypothesised that our long-range connectivity index could capture alterations in distant connections, thus confirming impaired integration in patients with 22q11DS. Indeed, we have shown reduced proportion of long-range connections in these patients in the bilateral superior frontal and parietal cortex, left anterior and dorsal cingulate cortex and right medial orbitofrontal cortex. Interestingly, these brain regions are involved in high order cognitive functions. Furthermore, in our previous study [79] we found reduced structural connectivity between nodes of the central executive network, a fronto-parietal network involved in cognition and executive functions. Altered cognitive functions have been reported in patients with 22q11DS, and we argue that these cognitive impairments may be related to underlying alterations in connectivity patterns. For instance, disrupted long-range connectivity in the inferior parietal cortex may be responsible for the impairments in mathematics and learning abilities observed in patient with 22q11DS [40]. Furthermore, dysconnectivity of the superior frontal and anterior cingulate cortices may be associated with psychotic symptoms, as showed by our previous studies investigating functional connectivity [80], variability of the blood oxygen-level dependent signal [81], structural connectivity [82], and structural covariance of cortical thickness [83]. However, our results reporting group differences in the long-range CI should be taken with caution. As reported in Supporting Information (S1 and S2 Figs), the reliability of our long-range CI was poor in some brain areas. A strong overlap was evident between the cluster of increased long-range CI in the patients on the right hemisphere Fig 7 and a region of poor reliability of the long-range CI. However, the same was not true for the other clusters showing significant group differences. Furthermore, we showed that this poor reliability was related to the accuracy of tractography, and not on the way our indices are computed. Despite the low accuracy of tractography reconstructions is a known issue, addressing it was beyond the aim of this study. However, we also found that the long-range connectivity index was highly variable between subjects. Therefore, this may have prevented us from showing exhaustive results in how the proportion of long-range fibers is affected in patients with 22q11DS.

Our results showed reduced short-range connections in patients with 22q11DS in regions including the inferior precentral gyrus bilaterally, the left precuneus, calcarine and paracentral/posterior cingulate cortices. Studies conducted in patients with polymicrogyria showed reduced proportion of short range-connections, presumably caused by altered laminar organization and reduced number of neurones that would be associated to impaired axonal connections [58]. Several findings converged in showing reduced cortical gyrification in patients with 22q11DS in widespread brain areas [53–57]. Among these investigations, two used a local gyrification index [55, 57] and identified with high resolution specific regions of reduced folding patterns in 22q11DS. These clusters were located in the bilateral pre- and post-central gyri, posterior cingulate gyrus, orbitofrontal, medial and middle frontal cortex, parieto-temporal junction, right supramarginal and superior temporal gyri, left occipital pole. In the present study, we observed in the same population of patients a decreased proportion of short-range tracts in the pre- and post-central and posterior cingulate cortices (Fig 6), thus preliminarily pointing to a concomitant alteration of gyrification and proportion of short tracts in these regions. The advantage of the connectivity index described in this study is that, as the local gyrification index, it is expressed at each cortical vertex and therefore, the two measures can be directly compared, giving a precise measure of concomitant alteration of cortical folding and connectivity that may indicate an altered developmental process occurring already during early embryonic stage. Indeed, it has been proposed that cortical folding rely on the tension exerted by the white matter fibers during the embryonic maturation of neural structures [84]. Therefore, additional investigations would be necessary to show if the reduction in short-range connections is directly associated to reduced gyrification in patients with 22q11DS.

This work bears some limitations, principally due to the weaknesses of DTI acquisitions and deterministic tractography algorithms for the reconstruction of the white matter streamlines [85]. Indeed, DTI methods cannot resolve multiple fibers’ orientations within the same voxel, thus being unable to accurately reconstruct streamlines in regions where crossing fibers are present [86]. However, this limitation was partially solved by the use of two tensors tractography for the reconstruction of the white matter tracts. Furthermore, our algorithm can be applied with other acquisitions (such as DSI) or other tractography techniques (e.g. probabilistic tractography) that allow a more reliable reconstruction of such complexes fibers’ bundles. Also, one could argue that we loose important information about the specific location of the fibers’ extremities when displaying measures of mean length, or of short- or long-range connectivity on the cortex. While this is certainly true, this limitation also applies to some extent to voxel-based measures of FA, RD or AD. However, our approach uses reconstructed streamlines where the information about the extremities can be retrieved and displayed for vertices of interest, as we showed in Fig 5. We thus felt that reducing the inherent multidimensional nature of the connectivity data by integrating the length information for only one of the fibers’ extremity was an innovative manner to quantify white matter connectivity, that has the main advantage of bringing the connectivity information in the cortical surface space for further integration with other cortical metrics. Finally, another possible artefact of the current algorithm is the fact that, as we consider fibres within a radius of 5mm, the same streamline may contribute to both sides of a gyrus. However, as showed in Supporting Information (S4 Fig), the geometry of the fibers ending in sulcal and gyral regions does not differ.

## Conclusions

To conclude, in this study we presented an algorithm to map the white matter pathways to the cortical surface and quantify white matter connectivity patterns over the cortex using measures of fibers’ length. In a sample of patients with 22q11DS, we revealed impairments in short- and long-range connections, which mirror previously reported dysfunctional integration in the syndrome and might represent a biological substrate for cognitive difficulties and psychotic symptoms typically observed in individuals with the syndrome. Finally, we suggested that these measures can be used together with measures of cortical morphology to understand the relationship between altered brain development and underlying connectivity in individuals with typical development or with neurodevelopmental diseases.

## Supporting information

S1 Fig Intra Class Correlation for the short- and long-range connectivity index (CI). (DOCX)

S2 Fig Coefficient of variation for the short- and long-range connectivity index (CI). (DOCX)

S3 Fig Proportion of white matter tracts ending in sulci or gyri in control participants (left) and in patients with 22q11DS (right). The number of terminations is a weighted value normalized by the length of the streamlines.
(DOCX)

S4 Fig Angles between vertices’ normal and fibers terminating in sulci and gyri. (DOCX)

S5 Fig White matter tracts connecting the clusters of significant difference in the mean tract length. Panel A) and B) indicate the left and right hemispheres respectively. The left and right columns display the fibers starting from the clusters of significant difference in mean path length in one control (left column) and one patient with 22q11DS (right column). The column in the middle shows the clusters of significant difference in mean path length in patients with 22q11DS compared to controls. The figures show that in the clusters where the mean path length is reduced in patients the density of long fibers connecting that cluster is reduced as well. On the opposite, in correspondence of the clusters of increased average path length in the patients the density of long fibers in higher than in controls.
(DOCX)
