# Supplementary material for: Quantifying indices of short- and long-range white matter connectivity at each cortical vertex
Source: PLoS One. 2017 Nov 15;12(11):e0187493. doi: 10.1371/journal.pone.0187493 (PMC5687731; doi:10.1371/journal.pone.0187493)
Supplement: S1 Fig — (DOCX) [file pone.0187493.s001.docx]

Reproducibility tests have been performed using the Intra Class Correlation (ICC).

First, we verified that the computation of our indices yielded similar results when using the same set of reconstructed fibers and re-computing uniquely the short and long-range CI. This analysis was performed in 10 control subjects and for each vertex of the cortical surface. We found that the Cronbach's alpha was = 1 for both the short- and long-range connectivity index and for every vertex in the cortical surface, thus indicating a perfect consistency.

To further test if the consistency of our indices was associated to the accuracy of fibers tracking, we performed an additional analysis including the same 10 controls subjects as for the previous one, for which a different DTI sequence was available, with the same parameters and acquired during the same scanning session. The brain maps reported in supplementary S1 Fig show the value of the Cronbach's alpha at each cortical vertex with a color code ranging from dark red (Cronbach's alpha ≤ 0.4 indicating poor consistency) to green (Cronbach's alpha ≥ 0.7 indicating good consistency). As evident from the brain maps, the short-range connectivity index showed a high ICC, with 84% of vertices having a good consistency. For the long-range connectivity index, the 70% of vertices showed good consistency, indicating that the reliability was good for most of the regions, but that in some brain areas the index was less reliable.


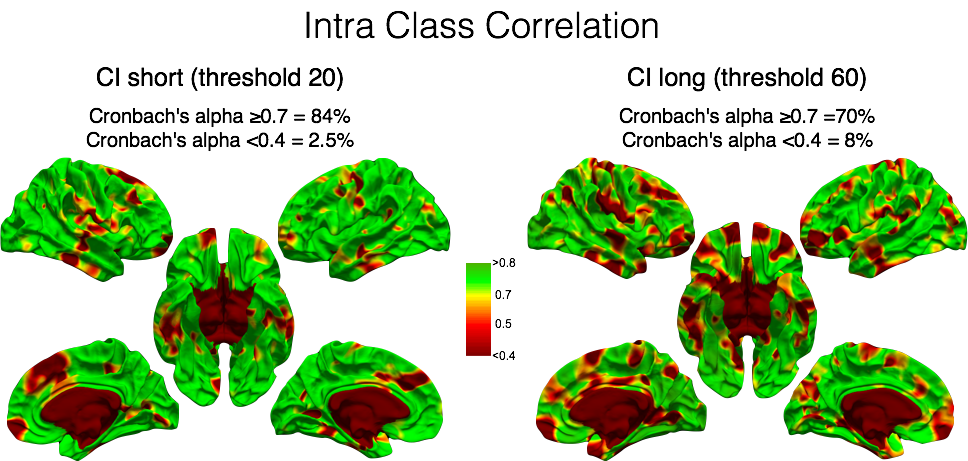


**S1 Fig. Intra Class Correlation for the short- and long-range connectivity index (CI).**
