# Supplementary material for: Quantifying indices of short- and long-range white matter connectivity at each cortical vertex
Source: PLoS One. 2017 Nov 15;12(11):e0187493. doi: 10.1371/journal.pone.0187493 (PMC5687731; doi:10.1371/journal.pone.0187493)
Supplement: S2 Fig — (DOCX) [file pone.0187493.s002.docx]

Inter-subject variability was performed using the Coefficient of Variation (CoV), measured as (SD/mean) *100. As for the ICC, it was computed for the short-range and long-range connectivity index and for each vertex of the cortical surface for all the patients and controls included in the study. A coefficient of variation of ≤10% indicates a small variability. Brain maps reporting the CoV for each cortical vertex are reported in S2 Fig. The short-range connectivity index showed a small variability throughout the cortical surface. However, the long-range connectivity index showed a high variability.


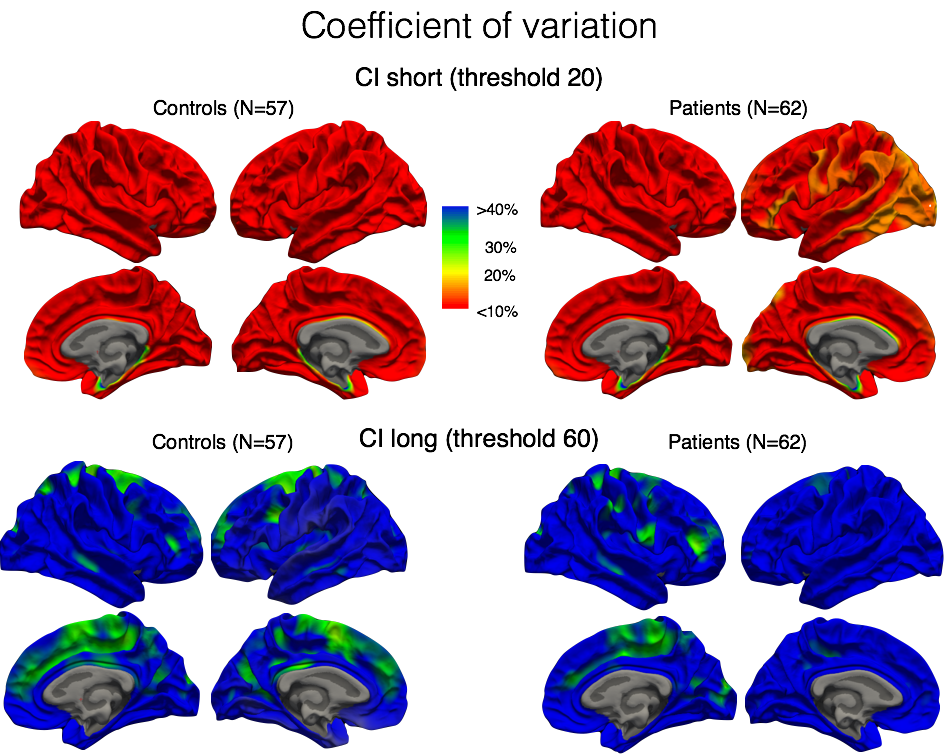


**S2 Fig. Coefficient of variation for the short- and long-range connectivity index (CI).**
