# Supplementary material for: Quantifying indices of short- and long-range white matter connectivity at each cortical vertex
Source: PLoS One. 2017 Nov 15;12(11):e0187493. doi: 10.1371/journal.pone.0187493 (PMC5687731; doi:10.1371/journal.pone.0187493)
Supplement: S3 Fig — The number of terminations is a weighted value normalized by the length of the streamlines. (DOCX) [file pone.0187493.s003.docx]

To assess if the terminations of the reconstructed streamlines were preferentially located in sulcal or gyral regions, we first localized their extremities on the cortical surface as described in the paragraph ‘Connectivity measures in the cortical surface’ in the method section. We then used the curvatures derived from the FreeSurfer processing pipeline to label each cortical vertex with a positive value, if it belonged to a sulcus, and a negative value if it belonged to a gyrus. Finally, we verified whether each termination corresponded to a sulcal or gyral vertex. Statistical comparison between the number of terminations in sulci and gyri was conducted with a Mann–Whitney U test.

S3 Fig show that in both control participants and patients with 22q11DS, the number of terminations ending in gyral regions is significantly greater than the number of streamlines ending in sulcal regions (p<0.0001).

**S3 Fig. Proportion of white matter tracts ending in sulci or gyri in control participants (left) and in patients with 22q11DS (right).** The number of terminations is a weighted value normalized by the length of the streamlines.
