# Supplementary material for: Quantifying indices of short- and long-range white matter connectivity at each cortical vertex
Source: PLoS One. 2017 Nov 15;12(11):e0187493. doi: 10.1371/journal.pone.0187493 (PMC5687731; doi:10.1371/journal.pone.0187493)
Supplement: S4 Fig — (DOCX) [file pone.0187493.s004.docx]

We further computed the angle between the terminations of the streamlines and the normal to each vertex on the cortical surface. The localization of the streamlines’ extremities and the labelling of the cortical vertices as belonging to sulcal or gyral regions was performed as described in the supplementary file S3.

As showed in the plots below, the angles between streamlines’ terminations and vertices’ normal did not differ between sulcal and gyral regions (p=0.28).

**S4 Fig. Angles between vertices’ normal and fibers terminating in sulci and gyri.**
