# Supplementary material for: Quantifying indices of short- and long-range white matter connectivity at each cortical vertex
Source: PLoS One. 2017 Nov 15;12(11):e0187493. doi: 10.1371/journal.pone.0187493 (PMC5687731; doi:10.1371/journal.pone.0187493)
Supplement: S5 Fig — Panel A) and B) indicate the left and right hemispheres respectively. The left and right columns display the fibers starting from the clusters of significant difference in mean path length in one control (left column) and one patient with 22q11DS (right column). The column in the middle shows the clusters of significant difference in mean path length in patients with 22q11DS compared to controls. The figures show that in the clusters where the mean path length is reduced in patients the density of long fibers connecting that cluster is reduced as well. On the opposite, in correspondence of the clusters of increased average path length in the patients the density of long fibers in higher than in controls. (DOCX) [file pone.0187493.s005.docx]

**
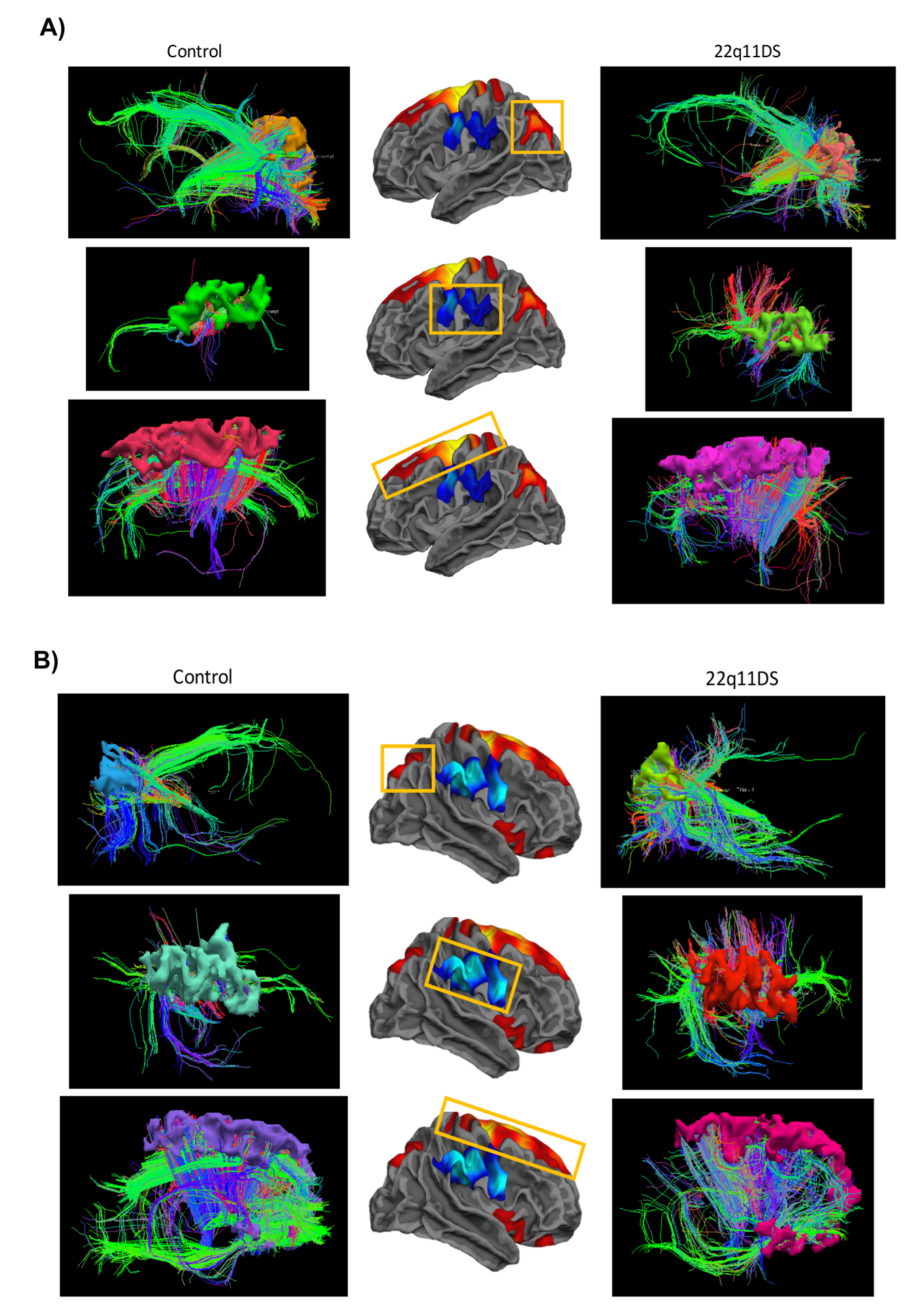
**

**S5 Fig. White matter tracts connecting the clusters of significant difference in the mean tract length.**

Panel A) and B) indicate the left and right hemispheres respectively. The left and right columns display the fibers starting from the clusters of significant difference in mean path length in one control (left column) and one patient with 22q11DS (right column). The column in the middle shows the clusters of significant difference in mean path length in patients with 22q11DS compared to controls. The figures show that in the clusters where the mean path length is reduced in patients the density of long fibers connecting that cluster is reduced as well. On the opposite, in correspondence of the clusters of increased average path length in the patients the density of long fibers in higher than in controls.
